# Region-specific tauopathy and synucleinopathy in brain of the alpha-synuclein overexpressing mouse model of Parkinson's disease

**DOI:** 10.1186/1471-2202-12-79

**Published:** 2011-08-03

**Authors:** Tiffany Kaul, Joel Credle, Thomas Haggerty, Adam W Oaks, Eliezer Masliah, Anita Sidhu

**Affiliations:** 1 Department of Biochemistry and Molecular and Cell Biology, University of California San Diego, La Jolla, California; 2 Department of Pathology, University of California San Diego, La Jolla, California

## Abstract

**Background:**

α-synuclein [α-Syn]-mediated activation of GSK-3β leading to increases in hyperphosphorylated Tau has been shown by us to occur in striata of Parkinson's diseased [PD] patients and in animal models of PD. In Alzheimer's disease, tauopathy exists in several brain regions; however, the pattern of distribution of tauopathy in other brain regions of PD or in animal models of PD is not known. The current studies were undertaken to analyze the distribution of tauopathy in different brain regions in a widely used mouse model of PD, the α-Syn overexpressing mouse.

**Results:**

High levels of α-Syn levels were seen in the brain stem, with a much smaller increase in the frontal cortex; neither cerebellum nor hippocampus showed any overexpression of α-Syn. Elevated levels of p-Tau, hyperphosphorylated at Ser202, Ser262 and Ser396/404, were seen in brain stem, with lower levels seen in hippocampus. In both frontal cortex and cerebellum, increases were seen only in p-Ser396/404 Tau, but not in p-Ser202 and p-Ser262. p-GSK-3β levels were not elevated in any of the brain regions, although total GSK-3β was elevated in brain stem. p-p38MAPK levels were unchanged in all brain regions examined, while p-ERK levels were elevated in brain stem, hippocampus and cerebellum, but not the frontal cortex. p-JNK levels were increased in brain stem and cerebellum but not in the frontal cortex or hippocampus. Elevated levels of free tubulin, indicating microtubule destabilization, were seen only in the brain stem.

**Conclusion:**

Our combined data suggest that in this animal model of PD, tauopathy, along with microtubule destabilization, exists primarily in the brain stem and striatum, which are also the two major brain regions known to express high levels of α-Syn and undergo the highest levels of degeneration in human PD. Thus, tauopathy in PD may have a very restricted pattern of distribution.

## Background

α-Synuclein (α-Syn) is a presynaptic, ubiquitously expressed protein in the brain, whose chief physiological function may be the regulation of synaptic levels of dopamine and other monoamines through modulation of the re-uptake function of monoamine transporters [1]. Overexpression of α-Syn, through its gene duplication and triplication, is linked to idiopathic Parkinson's disease [2-6], while its A30P and A53T mutants cause the autosomal dominant forms of familial PD [7,8]. In pathological states, α-Syn misfolds into aggregates and accumulates into neuronal inclusion bodies, termed Lewy bodies (LBs), which are pathological hallmarks of PD and other synucleinopathies [9-12]. Post-mortem immunohistochemical studies show the presence of hyperphosphorylated Tau (p-Tau), a protein normally linked to the genesis of Alzheimer's disease (AD), co-existing with α-Syn aggregates in the same neurons in PD and other synucleinopathies [13-20]. Conversely, in tauopathies such as AD, elevated levels of α-Syn have been found [21-26], along with LBs [21,27,28].

More recently, we have shown in the MPTP/MPP^+ ^*in vivo *and *in vitro *models of PD, respectively, that increases in α-Syn can initiate and sustain p-Tau formation with hyperphosphorylation at Ser202, Ser262 and Ser396/404, which are the same epitopes that are hyperphosphorylated in AD and lead to pathological changes [29-32]. In PD, there was an absolute requirement for the presence of α-Syn in the induction of hyperphosphorylation of Tau, and in MPTP-treated α-Syn-/- mice or toxin-treated neuronal cells lacking α-Syn we failed to observe any p-Tau formation [29,31], suggesting that α-Syn is central to tauopathy. In addition, we also found increases in levels of active GSK-3β [p-GSK-3β, hyperphosphorylated at Tyr216], a major kinase known to hyperphosphorylate Tau at the above mentioned sites. Blockade of this kinase with inhibitors prevent α-Syn-mediated p-Tau formation [29,31,32], thereby implicating p-GSK-3β in the mechanistic actions of α-Syn.

More recently, we found tauopathy in striatum of both the α-Syn overexpressing and the α-Syn A53T mutant mouse models of PD [33,34]. In both studies, we found Tau hyperphosphorylated at Ser202, Ser262 and Ser396/404, which was accompanied by elevated levels of α-Syn and p-GSK-3β. Interestingly, changes in p-Tau levels, which bind to and stabilize microtubules, lead to destabilization of the microtubule and actin networks in the striatum [33], indicating that p-Tau formation increased pathology in this brain region.

That tauopathy occurs in humans was confirmed using postmortem striata from PD patients, where elevated levels of α-Syn, p-Tau and p-GSK-3β were noted [35]. Interestingly, such changes were seen only in the striatum, but not in the inferior frontal gyrus of PD patients, where, although increased levels of α-Syn were noted, no changes in levels of p-Tau or p-GSK-3β were observed. This suggested that unlike AD, where tauopathy is seen throughout the brain, tauopathy in PD may be limited to dopaminergic neurons and have a more restricted pattern of distribution in the brain.

To further test this, the current studies were undertaken, whereby different brain regions of the α-Syn overexpressing mouse model of PD [36] were analyzed for changes in α-Syn, p-Tau and Tau kinases; the brain regions analyzed were brain stem, hippocampus, frontal cortex and cerebellum. The data presented here show that high levels of tauopathy, along with increases in α-Syn, are observed in brain stem, followed by more modest changes in hippocampus. In other brain regions tested, increases were seen only in p-Ser396/404 Tau. These tauopathic changes were independent of p-GSK-3β and appeared to be mediated by p-JNK and/or p-ERK.

## Results

### Expression levels of α-Syn in different brain regions of the PDGF-α-Syn overexpressing mice

We have previously [33] shown that α-Syn levels are significantly [*p *< 0.05] elevated [by 113% compared to litter-mate non-transgenic mice] in striata of PDGF-α-Syn overexpressing mice and that such changes were accompanied by profound tauopathy and increased levels of p-GSK-3β, the kinase known to hyperphosphorylate Tau at distinct epitopes. Increased levels of α-Syn were similarly observed in postmortem striata of PD and PD with dementia [35].

**Figure 1:**
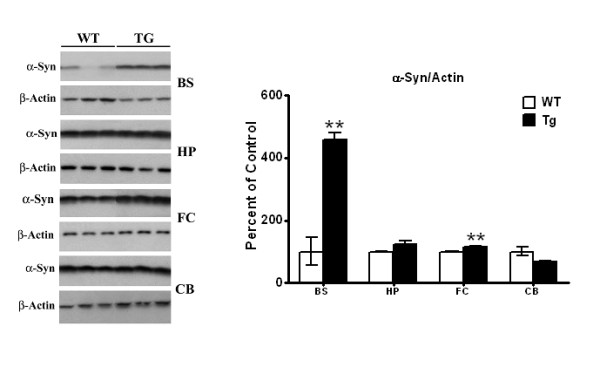
**Western blots of α-Syn levels in brain stem, hippocampus, frontal cortex, and cerebellum of PDGF-α-Syn overexpressing transgenic mice**. Brain stem [BS], hippocampus HP], frontal cortex [FC] and cerebellum [CVB] from PDGF-α-Syn transgenic mice and litter-mate non-transgenic mice [WT] were solubilized in RIPA buffer and analyzed by Western blots for α-Syn levels. α-Syn was normalized to β-actin. All values are expressed as percent change relative to changes observed in WT control animals. Results are from 3-4 animals per group; [*, *p *< 0.05] and [**, *p *< 0.01] compared to corresponding brain regions in WT animals. All blots are representative of samples.

To assess whether α-Syn levels were also increased in other brain regions of these transgenic mice, we examined brain stem, hippocampus, frontal cortex and cerebellum [Figure 1]. The highest levels of α-Syn were observed in brain stem, where [*p *< 0.01] increases of 358% were noted. The brain stem is to undergo neurodegenerative changes in Parkinson's disease [37], and such large increases in α-Syn levels in this region may parallel the pathological changes occurring in humans. More modest increases in α-Syn levels in hippocampus and frontal cortex were also noted, but they were significant only for the frontal cortex [15%, *p *< 0.01]. In the cerebellum, a small insignificant decrease of ~30% in α-Syn expression was seen [Figure 1].

### Tauopathic changes in different brain regions of the PDGF-α-Syn overexpressing mice

**Figure 2:**
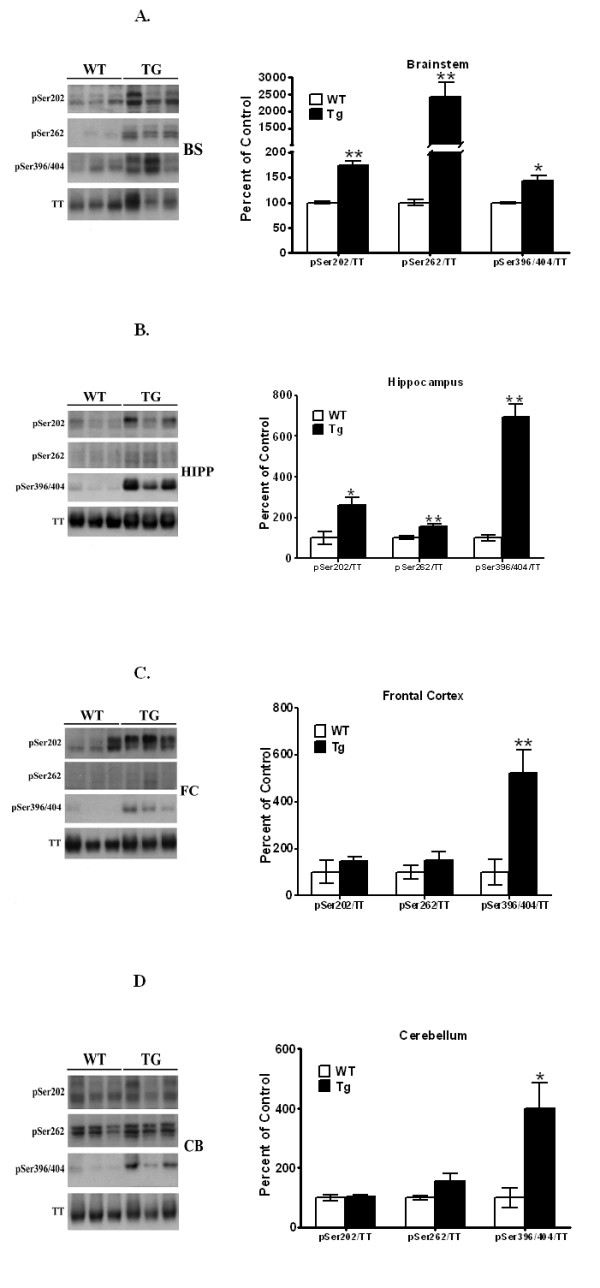
**Western blots of p-Tau levels in brain stem, hippocampus, frontal cortex, and cerebellum of PDGF-α-Syn overexpressing transgenic mice**. Brain stem, BS **[A]**, hippocampus, HP **[B]**, frontal cortex, FC **[C] **and cerebellum, CB **[D] **from PDGF-α-Syn transgenic mice and litter-mate non-transgenic mice [WT] were solubilized in RIPA buffer and analyzed by Western blots for p-Tau levels. The following p-Taus were normalized to total Tau: p-Ser202, p-Ser262, and p-Ser396/404. All values are expressed as percent change relative to changes observed in WT control animals. Results are from 3-4 animals per group; [*, *p *< 0.05] and [**, *p *< 0.01] compared to corresponding protein levels in WT animals. All blots are representative of samples.

In striata of the PDGF-α-Syn overexpressing mouse [33] as well as in striata of postmortem PD brains [35], high levels of tauopathy were previously detected where increases in p-Tau hyperphosphorylated at Ser202, Ser262 and Ser396/404 were observed. In the current study, the highest levels of tauopathy were seen in brain stem of the PDGF-α-Syn mice, followed by more modest tauopathic changes in other brain regions [Figure 2]. Thus, in the brain stem [Figure 2A], p-Ser202 levels of Tau were significantly [*p *< 0.05] increased by 73%, whereas p-Ser396/404 levels were elevated by 43%, compared to litter-mate nontransgenic mice. These increases in p-Ser202 are lower than those seen previously in the striatum [205% increase] of the transgenic mice, and the increases in p-Ser396/404 are also lower than those seen previously in striata, where we had observed a 50-fold (5000%) increase [33]. For p-Ser262, however, we observed a 25-fold (2500%) increase in brain stem, which was much higher than the 255% increase we had previously seen in the striatum [33].

In hippocampus [Figure 2B], significant increases were seen for all three epitopes of p-Tau, and although the levels of increases in p-Ser262 were much lower than those seen in brain stem, p-Ser202 and p-Ser396/404 were higher than in brain stem. Thus, increases of 158 [*p *< 0.05], 56 [*p *< 0.01] and 592% [*p *< 0.01] were seen for p-Ser202, p-Ser262 and p-Ser396/404, respectively, in hippocampus.

In frontal cortex [Figure 2C], no changes were seen for either p-Ser202 or p-Ser262, while p-Ser396/404 was significantly [*p *< 0.01] increased by 425%. Similarly in the cerebellum, we failed to observed changes in p-Ser202 and p-Ser262, while significant [*p *< 0.05] increases of 299% were noted for p-Ser396/404. Together, these data indicate that the tauopathy observed in the various brain regions has the following rank order of intensity: brain stem > hippocampus > frontal cortex > cerebellum.

### Immunohistochemical analysis of α-Syn and p-Tau

**Figure 3:**
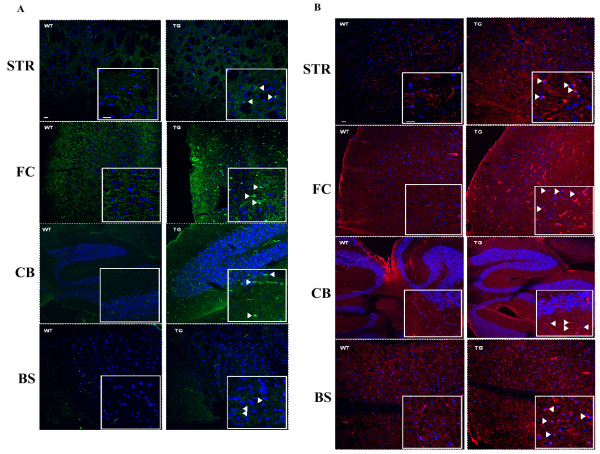
**Immunostaining of α-Syn and p-Tau in WT and Tg mice**. Immunohistochemical staining with (A) α-Syn, green, or (B) p-Tau, red, using specific antibodies and DAPI, blue, was conducted as described in Methods. The brain regions pictured top to bottom are: Striatum - Str, Frontal Cortex - FC, Cerebellum - CB, and Brain Stem - BS, in wild type (WT) left panel, and transgenic (Tg) right panel. Inset pictures show 4× magnified region with arrows indicating positive staining of large cellular aggregates. Scale bar: 40 u*M*, Inset Scale bar: *10 uM*.

Staining was performed on brain slices and the following brain regions were examined: striatum, frontal cortex, cerebellum and brain stem, using antibodies against α-Syn [Figure 3A] or p-Ser396/404 Tau [Figure 3B]. As was shown in the Western blots [Figure 1 &2], both α-Syn and p-Tau stain more intensely in the Tg mouse brains than wild type. In Figure 3A, white arrows indicate large cellular bodies staining positively for α-Syn in the Tg versus WT specific brain regions. A similar pattern of staining was seen for the specific brain regions stained for p-Ser396/404 Tau (Figure 3B) where white arrows indicate the presence of large cellular bodies seen in the Tg but not the WT brain sections. Moreover, the highlighted bodies shown in the inset pictures are reminiscent of the Lewy bodies commonly seen in Parkinson's disease brains.

### Tau kinases in different brain regions of the PDGF-α-Syn overexpressing mice

Tau is hyperphosphorylated by several kinases including p-GSK-3β, p-JNK, p-p38MAPK, p-ERK, cdk5, calmodulin kinase and protein kinase A [38]. Of these, we have previously found that p-GSK-3β was specifically activated by α-Syn *in vitro *in MPP+-treated SH-SY5Y cells and mesencephalic primary neurons [31,29] and *in vivo *in striata of MPTP-treated mice [32]. Moreover, blockade of p-GSK-3β with selective inhibitors or with lithium [31,32] abrogated GSK-3β activation and Tau hyperphosphorylation, suggesting a central role for this kinase in α-Syn-mediated p-Tau formation in dopaminergic cells and neurons.

**Figure 4:**
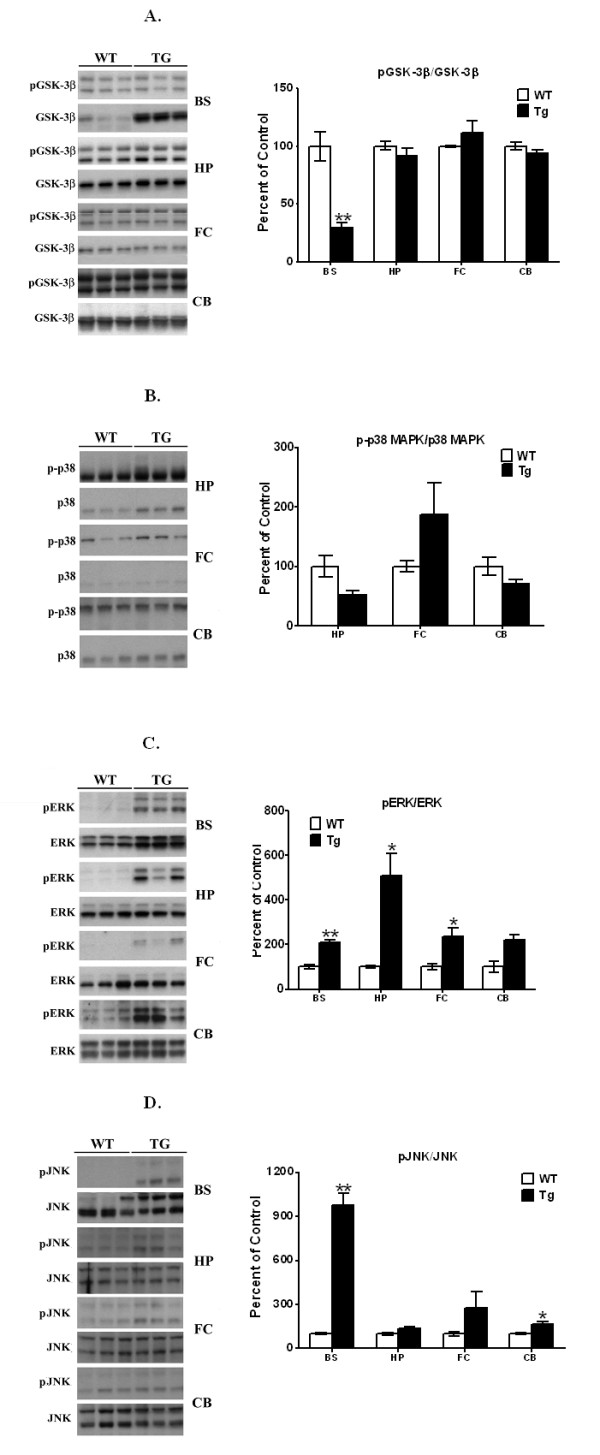
**Western blots of p-GSK-3β, p-p38, p-ERK, and p-JNK levels in brain stem, hippocampus, frontal cortex, and cerebellum of PDGF-α-Syn overexpressing transgenic mice**. Brain stem [BS], hippocampus [HP], frontal cortex [FC], and cerebellum [CB] from PDGF-α-Syn transgenic mice and litter-mate non-transgenic mice [WT] were solubilized in RIPA buffer and analyzed by Western blots for p-GSK-3β **[A]**, p-p38 **[B]**, p-ERK **[C]**, and p-JNK **[D]**. p-GSK-3β was normalized to GSK-3β, p-p38 to p38, p-ERK to ERK, and p-JNK to JNK. p-p38MAPK was not detected in brain stem and therefore this data is not shown. All values are expressed as percent change relative to changes observed in WT control animals. Results are from 3-4 animals per group; [*, *p *< 0.05] and [**, *p *< 0.01] compared to corresponding protein levels in WT animals. All blots are representative of samples.

In the present study, we examined activated p-GSK-3β levels [hyperphosphorylated at tyrosine 216] in the different brain regions. The antibody we used recognizes both the α-p-GSK-3β [upper band] and the β-p-GSK-3β band of the kinase. Therefore, in all studies, only the lower band was used in all calculations. In striata, we had previously found p-GSK-3β levels were significantly increased [by 112%, *p *< 0.05] in the PDGF-α-Syn transgenic mice [33]. In brain stem [Figure 4A], we failed to observe any increases in p-GSK-3β levels and instead found that p-GSK-3β was significantly [*p *< 0.01] decreased by 70%. The decrease was due to an overall increase in total GSK-3β levels [see Figure 4A], so that when p-GSK-3β levels were computed according to total GSK-3β levels, an overall net decrease was seen. This was accompanied by increases in levels of the inactive form of GSK-3β, phosphorylated at Ser9, which leads to inactivation of GSK-3β [data not shown]. In hippocampus [Figure 4A], no significant changes in p-GSK-3β or total GSK-3β were found compared to litter-mate nontransgenic mice. Similarly, we also found no changes in p-GSK-3β levels in either frontal cortex or in cerebellum [Figure 4A]. These combined data suggest that unlike our findings in striata [33], where p-GSK-3β was found to hyperphosphorylate Tau, in other brain regions of the PDGF-α-Syn mouse, p-GSK-3β did not participate in p-Tau formation and was not activated by α-Syn.

We therefore tested other kinases, by focusing on the mitogen-activated protein [MAP] family of kinases. In all brain regions, we failed to see any activation of p-p38MAPK [Figure 4B], eliminating a role for this kinase in hyperphosphorylation of Tau; we were unable to detect this protein in the brain stem, even in nontransgenic mice. When p-ERK levels were examined, significant and robust increases in the levels of this enzyme were seen in brain stem [114%, *p *< 0.01], hippocampus [407%, *p *< 0.05] and frontal cortex [34%, *p *< 0.05], with no changes in the cerebellum. These data suggest that in other brain regions, p-ERK may play an essential role in the hyperphosphorylation of Tau.

When p-JNK levels were examined [Figure 4D], large increases in this kinase were seen in brain stem [874%, *p *< 0.01], with nonsignificant changes seen in the hippocampus [32%] and frontal cortex [180%]. Small but significant increases were also seen in the cerebellum [64%, *p *< 0.05]. These data suggest that in the brain stem, Tau may be hyperphosphorylated by both p-JNK and p-ERK, while in hippocampus and frontal cortex tauopathy may proceed entirely through p-ERK, but not through p-JNK, activation. In cerebellum, tauopathic changes may be mediated entirely through the action of p-JNK, but not p-ERK.

### Elevated levels of free tubulin in brainstem of transgenic mice

**Figure 5:**
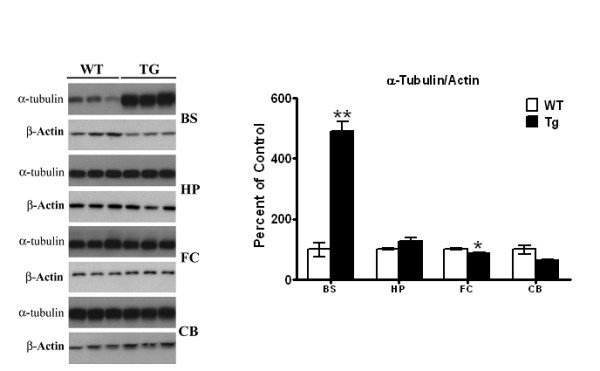
**Analyses of soluble tubulin in different brain regions**. Soluble fractions of RIPA extracts prepared from different brain regions of PDGF-α-Syn transgenic mice and wild type controls were analyzed for α-tubulin by Western blots as described in Methods. All values are expressed as percent change relative to changes observed in WT control animals. Results are from 3-4 animals per group; [*, *p *< 0.05] and [**, *p *< 0.01] compared to corresponding protein levels in WT animals. All blots are representative of samples.

Hyperphosphorylation of Tau at Ser262 reduces its affinity for microtubules, causing it to dissociate from the microtubular network, leading to destabilization of the microtubules [39,38]. In striata of the PDGF-α-Syn overexpressing transgenic, we have previously shown a significant increase in soluble tubulin levels of 13%, indicative of cytoskeleton remodeling in this brain region [33]. To test whether the tauopathy seen in the different brain regions of the transgenic mice also lead to microtubule remodeling, we measured levels of free tubulin in RIPA-soluble extracts [Figure 5]. In brain stem, we saw large and significant increases of 391% [*p *< 0.01] in tubulin levels in transgenic mice, compared to wild type controls. In other brain regions, no significant changes in tubulin levels were noted between wild type and transgenic mice, except in frontal cortex, where a small but significant [*p *< 0.05] decrease of 13% was noted. The higher levels of soluble tubulin seen in the brain stem parallels the higher levels of p-Ser262 Tau seen in this brain region compared to other regions, and suggests profound remodeling of the microtubular cytoskeleton.

## Discussion

We show here for the first time that an elevated state of tauopathy exists in the brain stem of the PDGF-α-Syn overexpressing mice, where high levels of p-Ser202, p-Ser262 and p-Ser396/404. This was accompanied by lower levels of tauopathy in the hippocampus. In cerebellum and frontal cortex, increases were seen for only p-Ser396/404, but not for p-Ser202 or p-Ser262. The severity of tauopathy, in general, is linked to parallel increases in α-Syn. Thus, in the brain stem, where we observed the most severe tauopathy, we also observed the highest increases in α-Syn levels. By contrast, other brain regions that showed lower increases in α-Syn levels were also characterized by lower levels of tauopathy. Our earlier studies have shown a tight linkage between α-Syn and p-Tau levels, and we have shown a mandatory requirement for α-Syn in tauopathy. Thus, in α-Syn knock-out animals or in cells lacking α-Syn [29], we fail to observe any tauopathic changes upon treatment with PD-inducing neurotoxins. In AD, it is long believed that the amyloid protein, β-amyloid, triggers the cascade of events that leads to generation of p-Tau. Similarly in the context of PD, emerging evidence from our laboratory has identified α-Syn to be the amyloid protein triggering tauopathy.

There have been only limited studies identifying the sites of hyperphosphorylation of Tau in PD brains. In one of these, Tau was found to be hyperphosphorylated at Ser396/404 in synaptic fractions from frontal cortex of PD postmortem striata [40]. Studies from our own laboratory have shown that Tau in striata of PD patients is hyperphosphorylated at Ser202, Ser262 and Ser396/404, where increases of 23, 34 and 81% were observed in PD compared to control, non-diseased striata [35], testifying to the pathophysiological relevance of these sites. There are more than 45 sites of hyperphosphorylation of Tau identified in AD [38] and the molecular consequences of only a few of these sites are known. Tau is primarily located along axons and, in general, hyperphosphorylation of Tau reduces its affinity for microtubules [MTs], leading to their destabilization, with eventual degeneration of neurons. Hyperphosphorylation at the microtubule MT binding domain [residues 244-368] of Tau is especially crucial in regulating MT stabilization, and phosphorylation at Ser262 detaches Tau from MTs, resulting in their destabilization [41]. Hyperphosphorylation at Ser396/404 promotes self assembly of Tau to form aggregates of Tau [39]. Moreover, *in vitro *studies using Tau peptides showed that phosphorylation of Tau at Ser262 and Ser356 modified both the negative charge and the local conformation near the phosphorylation sites, reducing the affinity of the peptides to bind to MTs [42]. Therefore, our finding of very high levels of p-Ser262 in the brain stem may explain the increased levels of soluble α-tubulin seen in this brain region. By contrast, the other brain regions had either no [frontal cortex and cerebellum] or low increases in p-Ser262 levels [hippocampus], and, therefore, also had no changes in soluble α-tubulin.

The pattern of tauopathy observed in this mouse model of PD closely parallels the Braak synucleinopathic staging scheme of Parkinson's disease initially proposed by Braak et al. [37]. Thus, it has been proposed that lesions initially occur in the glossopharyngeal and vagal nerves and in the anterior olfactory nucleus, progressing to the brain stem, and thereafter pursuing an ascending course of pathology [37]. Cortical involvement follows, beginning with the anteromedial temporal mesocortex, followed by the neocortex, especially the prefrontal region. The concept proposed by Braak and colleagues [37] that lower brainstem synucleinopathy represents "early PD", subsequently progressing within the human lifetime to involve the mesencephalon, suggests that the brain stem may be more severely affected than dopaminergic neurons, and this is borne out by the higher degree of tauopathy seen in the brain stem compared to the striatum in the PDGF-α-Syn overexpressing mice. Indeed, our findings of higher levels of soluble α-tubulin in the brain stem of the transgenic mouse as compared to the wild type also occurs in parallel with the higher levels of synucleinopathy seen in this brain region, suggesting that the brain stem is undergoing more degeneration than any other brain region tested.

Unlike our previous findings, the current study does not indicate any involvement of p-GSK-3β in the accumulation of p-Tau in brain regions other than the striatum. Previously, we had found that α-Syn could recruit and activate p-GSK-3β in a specific manner in the striatum [32,35] and that such activation of p-GSK-3β was dependent on autoxidation of dopamine [34]. The current study suggests that in non-dopaminergic neurons, other kinases may instead become activated. Thus, our data suggest that p-ERK and p-JNK, but not p-p38MAPK, become activated. To our knowledge, this is the first report of a possible involvement of p-ERK and p-JNK in the genesis of tauopathy in PD. Thus, increased levels of p-ERK are seen in brain stem, hippocampus and frontal cortex, but not in cerebellum, indicating the possible participation of this kinase in the first three brain regions mentioned. Indeed, in both PD striata and in cellular as well as animal models of PD, ongoing studies in our laboratory suggest an important and central role for p-ERK in the pathogenesis of PD [Duka & Sidhu, Unpublished Observations]. Since p-JNK is activated in cerebellum, it is likely that the increase in p-Ser396/404 seen here proceeds through p-JNK. By contrast, the increase in tauopathy seen in hippocampus is likely to occur entirely through p-ERK and not p-JNK, since p-JNK is not activated in this region. In brain stem, however, the high levels of both p-ERK and p-JNK may together contribute to the very high levels of tauopathy seen in this region. It should be noted, however, that our results do not eliminate the possibility that other kinases may also participate in hyperphosphorylation of Tau, either in concert with, or entirely independent of, p-ERK and p-JNK.

It is now well established that overexpression of α-Syn, through its gene duplication and triplication, is linked to idiopathic Parkinson's disease [43,9], although the mechanism[s] by which PD occurs in such populations remains elusive. The PDGF-α-Syn overexpressing mouse model closely mimics sporadic PD [36] and our data show that at least part of the mechanism may be due to tauopathic changes in not only the striatum of these mice [33] but also in other brain regions, such as the brain stem, hippocampus, frontal cortex and cerebellum. Therapy aimed at reducing the overall tauopathic burden may be especially useful in alleviating PD.

## Conclusion

In summary, our studies show that in the α-Syn overexpressing mouse model of PD, tauopathy occurs in parallel to synucleinopathy. In brainstem, which has the highest levels of α-Syn, the highest levels of tauopathy were also seen, which was accompanied by elevated levels of soluble α-tubulin. In other brain regions, lower levels of α-Syn also gave rise to lower levels of tauopathy, without any increases in α-tubulin. The major Tau kinase, GSK-3β, was not activated in any of the brain regions examined. Instead, activation of both p-ERK and p-JNK was variably seen implicating these kinases in the genesis of PD.

## Materials and methods

### Materials

The antibodies used in this study are: anti-Tau MAB361 from Millipore [Temecula, CA]; anti-Tau Neurofibrillary Tangles Marker AHB0042 and anti-Tau (p-S262), Biosource Invitrogen [Carlsbad, CA]; anti-α-Syn CAT# 610787, anti-GSK-3β CAT# 612313 and anti-p-GSK-3β [purified mouse anti-GSK-3β (pY216) CAT # 612313], from BD Transduction Labs [San Jose, CA]; anti-α-Tubulin T6074 from Sigma-Aldrich [St. Louis, MO]; anti-β-actin SC-1616 from Santa Cruz Biotechnology, Inc. [Santa Cruz, CA]; The CP-13 and PHF-1 [recognizing Tau-Ser202 and Tau-Ser396/404, respectively] were gifts from Dr. Peter Davies [New York]; GAPDH [14C10 #2118], p-ERK [Phospho-p44/42 MAPK (Erk1/2) (Thr202/Tyr204) (E10) #9106], ERK [p44/42 MAPK (Erk1/2) (137F5) #4695], p-p38 [Phospho-p38 MAPK (Thr180/Tyr182) (28B10) #9216], p38 MAPK [#9212], p-SAPK/JNK [Phospho-SAPK/JNK (Thr183/Tyr185) (G9) #9255], and SAPK/JNK [#9252] were all from Cell Signaling Technology [Danvers, Massachusetts].

### Animals

All studies with animals were conducted under strict guidelines of the National Institutes of Research and were approved by Georgetown University Animal Care and Use Committee. Hemizygous mice overexpressing α-Syn driven by the platelet-derived growth factor [PDGF] promoter were imported (from E. Masliah, University of California San Diego, CA). For all experiments, hemizygous PDGF-α-Syn mice were bred with wild type (WT) mice (C57BL/6 × DBA/2 F_1_; B6D2F1/J) obtained from Jackson Labs to produce both WT and PDGF-α-Syn littermates, and a breeding colony was established as described previously [36].

### Western Blot Analysis

Western blot analysis was performed as described elsewhere [32,35]. Briefly, mouse brain stem, hippocampus, frontal cortex, and cerebellum from 11 month old WT and Tg animals were homogenized in RIPA buffer (50 mM Tris-HCl pH 7.5, 150 mM NaCl, 1 mM EDTA) containing 0.5% Triton X-100, 0.5% sodium deoxycholate, and 0.1% SDS in the presence of protease inhibitor cocktail tablets (Complete Mini, EDTA-free; Roche Diagnostics GmbH, Germany) and phosphatase inhibitor cocktail (Halt™ Protease Inhibitor Cocktail; Pierce). Lysates were inverted at 4°C for 30 min, followed by centrifugation for 10 min at 15,000 × g and 4°C. Supernatants were collected and protein concentrations were measured using the MBLowry assay. Samples were analyzed by Western blots on 10-20% Tris-HCl Criterion gels (Bio-Rad), after blocking with 20 mM Tris-buffered saline, pH 7.6 containing 0.1% Tween 20 (TBST) and 5% (wt/vol) blotting grade blocker non-fat dry milk (Bio-Rad) for 1 hour at room temperature. Western blots were developed with a wide range of specific human Tau antibodies that recognize the protein at different phosphorylation sites, including: CP13 (p-Ser202) (1:500), PHF-1 (p-Ser396/404) (1:1000) and p-Ser262 (1:500). Total Glycogen Synthase Kinase-3β (GSK-3β) was probed for with mouse GSK-3β antibody (1:500) and phospho-GSK-3β was probed for using mouse phospho-specific (pY216) antibody (1:500). α-Tubulin levels in RIPA-solubilized lysates was probed for with mouse α-tubulin antibody (1:4000). To probe for α-Syn, samples were run on 10-20% Tris HCl Criterion gels (Bio-Rad) and immunoblotted with mouse α-synuclein (1:1000) antibody. All proteins were normalized to total Tau (1:1000), β-actin (1:500) or GAPDH (1:1000). After incubation for 2 hours at room temperature with HRP-conjugated secondary antibodies (1:3000, Santa Cruz), proteins were revealed by enhanced chemiluminescence (Perkin Elmer). Images were scanned by Scanner EPSON Perfection V700 Photo and then quantified using ImageJ.

### Immunohistochemistry

IHC analysis of mouse brain coronal sections was performed as previously described [33], with slight modifications. Briefly, mouse brains from 11 month old control wild-type and age-matched Tg α-Syn mutant mice were perfused with 4% PFA, and prepared in a sequential sucrose gradient, from 10% to a final 30% sucrose soak. Sections (5 μm) were washed, permeabilized, and stained in the following manner. Each slice was washed 3 times in 1 mg/ml NaBr_2_, 1 × PBS pH 7.4, for 5 min at room temperature. Following the NaBr_2 _auto-fluorescence quenching treatment, each slice was washed 6×, for 10 min in 1 × PBS pH 7.4, 1% Triton X-100 followed by blocking for 1 hr at room temperature in 1 × PBS pH 7.4, 1% Triton X-100, 10% FCS. Incubation with primary antibody occurred at 4°C, overnight in the dark, in blocking buffer using the following concentrations of antibodies: anti-α-Syn, 1:750; PHF-1, 1:500. Following staining, each slice was washed 3 × in 1 × PBS pH 7.4, 1% Triton X-100 at room temperature, incubated for 30 min in blocking buffer, and washed a final 3× in 1 × PBS pH 7.4, 1% Triton X-100 before incubation with appropriate secondary antibody; goat anti-mouse alexa-fluor 594, goat anti-mouse alexa-fluor 488, at the a concentration of 1:500. Following staining with secondary antibody, each slice was washed 3 × in 1 × PBS pH 7.4, 1% Triton X-100 at room temperature. Stained slices were mounted to Fischer Scientific Superfrost standard microscope slides using Fluoromount-DAPI. Fluorescence images were captured using a laser scanning confocal microscope (Olympus FV300). Paired images between WT and Tg tissue for all figures were collected at the same laser power, gain, and offset settings. Post-collection processing was performed using ImageJ and applied uniformly to all paired images.

### Statistical Analysis

Results were expressed as mean ± S.E.M. and statistically analyzed by the Student's t-test between two groups. Statistical significance was accepted at the [*p *< 0.05] level.

## Competing interests

The authors declare that they have no competing interests.

## Authors' contributions

TK performed majority of the experiments, collected data and performed statistical analyses; JC performed the IHC studies, conducted the interpretation and assisted in preparation of manuscript; TH performed the tubulin studies, analyzed data, statistics and helped in interpretation, we well as writing of the manuscript; AWO conducted the dissections of various brain regions and assisted in interpretation of results and writing of the manuscript; EM provided the transgenic mice and assisted in interpretation of data; AS conceived of the study, and participated in its design and coordination. All authors read and approved the final manuscript.
